# Synthesis of Multifunctional Oligomethylsilsesquioxanes by Catalyst-Free Hydrolytic Polycondensation of Methyltrimethoxysilane under Microwave Radiation

**DOI:** 10.3390/polym15020291

**Published:** 2023-01-06

**Authors:** Alexandra A. Kalinina, Olga B. Gorbatsevich, Nikita G. Yakhontov, Nina V. Demchenko, Nataliya G. Vasilenko, Valentina V. Kazakova, Aziz M. Muzafarov

**Affiliations:** 1 Enikolopov Institute of Synthetic Polymeric Materials, Russian Academy of Sciences, 117393 Moscow, Russia; 2 A. N. Nesmeyanov Institute of Organoelement Compounds, Russian Academy of Sciences, 119991 Moscow, Russia

**Keywords:** catalyst-free hydrolytic polycondensation, alkoxysilanes, methyltrimethoxysilane, polyorganosilsesquioxanes, microwave radiation

## Abstract

The catalyst-free hydrolytic polycondensation of methyltrimethoxysilane under microwave radiation has been studied. The effect of molar ratios of the reagents (MTMS/H_2_O = 1/0.5–1/9), radiation power (20–300 W), temperature (30–50 °C) and duration of exposure (2.5–90 min) on the course of the process is considered. It has been shown that the use of microwave radiation promotes the activation of the process, and almost complete conversion of the monomer can be achieved in 5 min at 30 °C, 20 W and an MTMS/H_2_O ratio of 1/3. The optimal radiation power for the maximum conversion of the monomer and MeO-groups is in the range from 20 to 100 W. An increase in the water amount, the duration and temperature of the process contribute to an increase in the monomer conversion, a decrease in the content of residual MeO-groups and the yield of non-volatile oligomethylsilsesquioxanes. The limits of this approach using to the synthesis of multifunctional branched polyorganosilsesquioxanes are determined. Depending on the process conditions, homogeneous water–alcohol solutions of oligomethylsilsesquioxane with a concentration of 20 to 50 wt.% can be obtained. The OH-group content and the molecular weight of the obtained oligomers vary from 10 to 30 wt.% and from 1000 to 600 Da, respectively.

## 1. Introduction

One of the most important representatives of organosilicon polymers are polyorganosilsesquioxanes, the properties and applications of which are determined by their molecular parameters. Thus, high molecular weight polycyclic polyorganosilsesquioxanes are traditionally used as binders [1,2,3]. Organosilsesquioxanes insoluble in organic solvents are used as sorbents and fillers [4,5,6,7,8]. Low molecular weight and polyhedral silsesquioxanes are effective as chain structural elements or molecular additives that increase the thermal stability and strength of composites [9,10,11,12,13].

The development of synthetic approaches that make it possible to control the structure of the resulting products has led to the emergence of new comb-shaped and star-shaped siloxanes with a silsesquioxane backbone [14,15], hyperbranched polyorganosiloxanes and their transformation into nanogels with a controlled core–shell ratio [16], cyclosiloxane polyols [17], heat-resistant and strong ladder silsesquioxanes with a controlled number of defective bonds [18,19,20,21].

Soluble highly functional organosilsesquioxanes are of particular interest. The complexity of obtaining such structures by traditional approaches, such as hydrolytic polycondensation of organotrichlorosilanes [1,22] and catalytic hydrolysis of organotrialkoxysilanes [3,23,24], is due to high degree of condensation of the products, caused by the high reactivity of the monomer, and the formation of hydrochloric acid during the reaction in the first case and the using of catalysts in the second [9].

Obviously, to prevent this undesirable process from occurring, it is necessary to carry out the reaction under neutral conditions. However, the hydrolytic polycondensation of alkoxysilanes under neutral conditions is characterized by the presence of an induction period [25], the duration of which depends on the structure of the starting reagent, and is probably due to the different surface-active properties of the starting monomers and hydrolysis intermediates [26].

Previous studies on the intensification of the hydrolytic polycondensation of alkoxysilanes under neutral conditions, within the framework of the concept of chlorine-free chemistry of silicones, showed the potential of carrying out these reactions under pressure [27,28,29,30,31,32] and also using ultrasonic radiation [33]. In both cases, the processes were carried out in the absence of solvents and catalysts, and were characterized by a significant shortening in the duration of the reactions, up to 5 min in the case of ultrasonic radiation. The use of such approaches in the case of methyltrialkoxysislanes led to the formation of stable aqueous–alcoholic solutions of polyhydroxyfunctional oligomethylsilsesquioxanes with a controlled content of hydroxyl groups and molecular weight characteristics. Such products may be of interest as water repellents and thin coatings for various materials [34,35,36,37].

That is, the methods of intensification of hydrolytic condensation processes not only allow the simplification the technological aspects of the synthesis of organosilsesquioxanes, while creating prerequisites for improving the environmental and economic performance of processes, but also lead to the production of new products with new areas of application. These results confirm the promise of studying of the effect of other physical fields, in particular, microwave radiation (MW radiation), on the hydrolytic polycondensation processes of alkoxysilanes under catalyst-free conditions. The use of this activation method seems logical and justified exactly in the processes of hydrolytic polycondensation, since in this case, one can expect the activation of the main reagent of the process- water molecules, due to MW radiation.

The data available in the scientific literature show that the use of MW radiation has a noticeable effect on the processes involving siloxane compounds, including alkoxysilanes. For example, the use of MW radiation during alkaline hydrolysis of methyltrimethoxysilane (MTMS) in diglyme makes it possible to obtain cubic octamethylsilsesquioxane in the form of solid particles in good yield (76%) due to the formation of micelles from eight molecules of MTMS in diglyme [38]. The use of MW radiation during the acid hydrolysis of tetramethoxysilane leads to the formation of a sol of silica nanoparticles with a textured surface, which is not typical for silica sols obtained by the Stöber method, which have a smooth surface [39]. The use of MW radiation as a heat source has been successfully used in the preparation of hybrid silica nanoparticles of silicon dioxide modified with 3-chloropropyltrimethoxysilane [40].

It was also shown that a rapid covalent modification of silicon oxide surfaces by alcohol-containing compounds is observed under the influence of MW radiation [40]. However, there are no data on the effect of MW radiation on the processes of hydrolytic polycondensation under neutral conditions. Therefore, the aim of this work is to study the catalyst-free hydrolytic polycondensation of MTMS under MW radiation (MWHP). An important task is to study the influence of the ratios of the starting reagents, temperature, process duration, and radiation power on the structure and composition of the resulting products.

## 2. Materials and Methods

All used reagents are commercially available: MTMS, chlorodimethylvinylsilane (ABCR, Karlsruhe, Germany), methyl-tert-butyl ether, pyridine (ECOS-1, Moscow, Russia). All reagents were purified by standard methods [41]: MTMS, chlorodimethylvinylsilane, methyl-tert-butyl ether by atmospheric distillation; pyridine was purified and dried by distillation over CaH_2_.

Gas–liquid chromatography (GLC) was performed on a Khromatek-Analitik-2000M chromatograph (Yoshkar-Ola, Russia) with a katharometer detector. The column length was 2 m and the diameter was 3 mm. Helium was used as the carrier gas; the gas rate was 30 mL/min. SE-30 (5%) deposited on Chromaton-H-AW was used as the stationary phase. 

Gel-permeation chromatography (GPC) was performed on a chromatographic system consisting of a STAIER series II high-pressure pump (Aquilon, Nakhodka, Russia), a RIDK 102 refractometric detector (Czech Republic), and a JETSTREAM 2 PLUS column thermostat (KNAUER, Berlin, Germany). The temperature was controlled at 40 °C (±0.1 °C). Tetrahydrofuran was used as the eluent, the flow rate was 1.0 mL/min. A 300 × 7.8 mm column filled with Phenogel sorbent (Phenomenex, Torrance, CA, USA), particle size 5 μm, pore size 103 A was used (passport separation range—up to 75,000 D). Recording and processing of data was carried out using UniChrom 4.7 software (Minsk, Belarus). 

Proton nuclear magnetic resonance (^1^H NMR) spectra were recorded on a Bruker AC-250 spectrometer using CDCl_3_ as the solvent. 

Infrared (IR) spectra were recorded using a Nicolet iS50 IR spectrometer with an ATR set-up (diamond) with a resolution of 4 cm^−1^ at 32 scans. 

Gas chromatography–mass spectrometry (GC–MS) spectra were recorded at Moscow State University (Moscow, Russia). GC–MS was performed with a Shimadzu GCMS-QP2010 Ultra.

Elemental analysis was carried out at the A.N. Nesmeyanov Institute of Organoelement Compounds of the Russian Academy of Sciences (INEOS RAS, Moscow, Russia).

MWHP of MTMS procedure:

Samples were irradiated in a MW oven CEM Discover System Model 908,010 (Matthews, NC, USA), equipped with a programmable pressure and temperature controller. Temperature control was carried out using an IR sensor built into the microwave oven, with its preliminary calibration relative to a mercury thermometer. Samples of MTMS and the calculated amount of water were placed in a round-bottom flask with a reflux condenser, based on the required molar ratio of the reagents, subjected to MW irradiation for a specified time at a starting pressure and a given temperature. Reaction conditions, reagent ratio and results of the analysis are shown in Table 1.

Hydrolytic polycondensation of MTMS by stirred at 500 rpm procedure:

MTMS and water in molar ratio of 1/3 were added in a flask and was stirred at 500 rpm at 30 °C for 90 min. The conversion of MTMS was monitored by GLC.

The blocking of MTMS MWHP products was carried out according to the well-known method described in [31]. GC–MS spectrum of the low molecular weight fraction of the blocked product: 171.0 [Si_2_C_7_H_15_O]^+^; 191.0 [Si_2_C_6_H_15_O_3_]^+^; 261.1 [Si_3_C_9_H_21_O_3_]^+^; 331.1 [Si_4_C_12_H_21_O_3_]^+^; 351.1 [Si_4_C_11_H_27_O_5_]^+^. IR spectrum of the oligomeric fraction of the blocked product (ATR set-up (diamond)), ν/cm^−1^: 3051 w, 2961 (C–H) w; 1595 (C=C) cl; 1406 (C=C) w; 1267 w, 1252 (Si–C) w; 1029 s, 954 (Si–O) w; 835 m, 780 s, 704 w, 518 (Si–C) w. ^1^H NMR spectrum of the oligomeric fraction of the blocked product (CDCl_3_, δ, ppm): 0.03–0.21 (m, SiMe); 3.48–3.53 (m, OMe); 5.70–6.21 (m, SiVin).

After keeping the reaction mixture of MTMS MWHP at room temperature for two months, the resulting gel was centrifuged three times with washing with MTBE from the remaining soluble oligomethylsesquioxanes. Then, it was dried in a vacuum oven at 50 °C/10 Torr during the day. IR spectrum of polymethylsilsesquioxane gel of the aged product (ATR set-up (diamond)), 3406 cm^−1^ (O–H) w; 2971 cm^−1^ (C–H) w; 1408 cm^−1^ w, 1268 cm^−1^ (Si–C) m; 1008 cm^−1^ w, 904 cm^−1^ m, 849 cm^−1^ (Si–O) m; 757 cm^−1^ w, 548 cm^−1^ (Si–C) m; 3406 cm^−1^ (O–H) w; 2971 cm^−1^ (C–H) w; 1408 cm^−1^ w, 1268 cm^−1^ (Si–C) m; 1008 cm^−1^ w, 904 cm^−1^ m, 849 cm^−1^ (Si–O) m; 757 cm^−1^ m, 548 cm^−1^ (Si–C) m. Elemental analysis: 18.44% C, 5.56% H, 39.50% Si.

## 3. Results and Discussion

**Figure 1:**
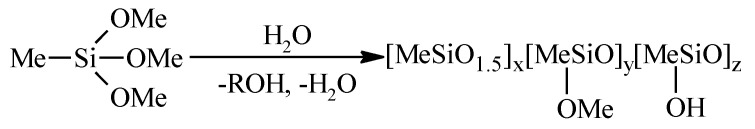
General scheme of the reaction for MWHP of MTMS.

**Figure 2:**
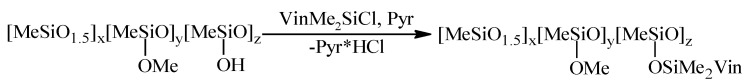
Scheme for the blocking reaction of the MTMS MWHP products.

**Figure 3:**
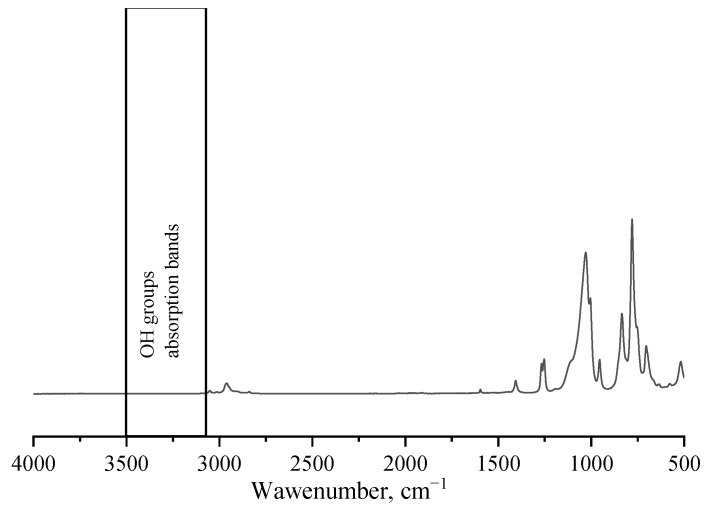
IR spectrum of blocked Sample 5 (Table 1).

The study of MTMS MWHP in the absence of solvents, regardless of the process conditions, showed that the process proceeds according to the classical scheme for hydrolytic polycondensation of trialkoxysilane, with the formation of soluble oligomeric products with the silsesquioxane structure (Figure 1):

To analyze the composition and stabilize the resulting products of the MTMS MWHP, their hydroxyl groups were blocked under conditions that did not disturb the structure of oligomeric products with chlorodimethylvinylsilane [28] (Figure 2):

The blocking efficiency was approved by the absence of IR spectra absorption bands in the region of 3600–3500 cm^−1^, characteristic of the valence vibrations of the OH-groups. As an example, the IR spectrum of Sample 5 (Table 1), typical of such products, is shown in Figure 3.

**Figure 4:**
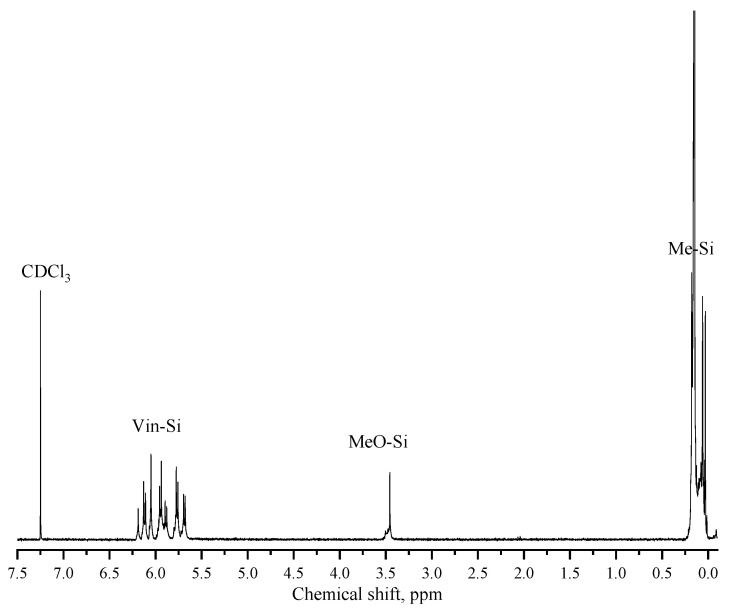
^1^H NMR spectrum of the blocked Sample 5 (Table 1).

The conversion of the starting monomer was determined by GLC from the MTMS content in the reaction mixture. The quantitative content of the residual methoxysilyl- and hydroxysilyl-groups in the oligomeric fraction of the blocked product was determined by ^1^H NMR spectroscopy from the ratio of the integral intensities of the MeSiO_1.5_-, MeOSi- and VinMe_2_SiO-group protons. As an example, Figure 4 shows the ^1^H NMR spectrum of the blocked Sample 5 (Table 1).

Blocked products of the MTMS MWHP were distillated at 1 Torr. A low molecular weight fraction with T_b_ ≤ 85 °C/1 Torr was isolated and analyzed by GLC and GC–MS, and an oligomeric fraction with T_b_ > 85 °C/1 Torr was analyzed by a combination of GC–MS, GPC, IR and NMR spectroscopy methods.

**Figure 5:**
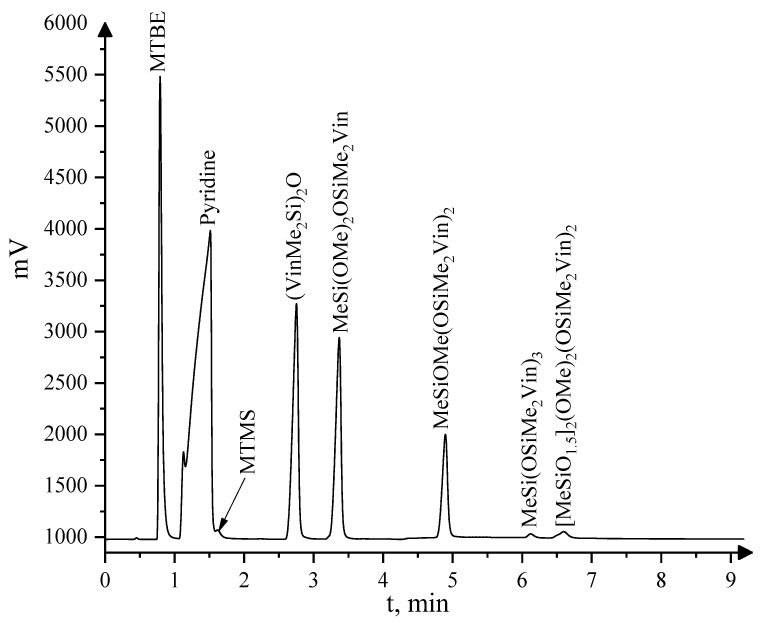
GLC curve for the fraction of Sample 5 (Table 1) with T_b_ ≤ 85 °C/1 Torr.

The low molecular weight fraction was a mixture of blocked mono- and dimeric products of the hydrolysis and condensation of the initial monomer according to the GLC data (Figure 5), as well as residues of the solvent, pyridine, tetramethyldivinyldisiloxane, and the initial MTMS. Due to the lack of standard samples, the GC–MS method was used for identifying siloxane products.

The oligomeric fraction is a mixture of highly functional soluble oligomers with a methylsilsesquioxane structure according to ^1^H NMR spectroscopy and GPC (Table 1).

**Figure 6:**
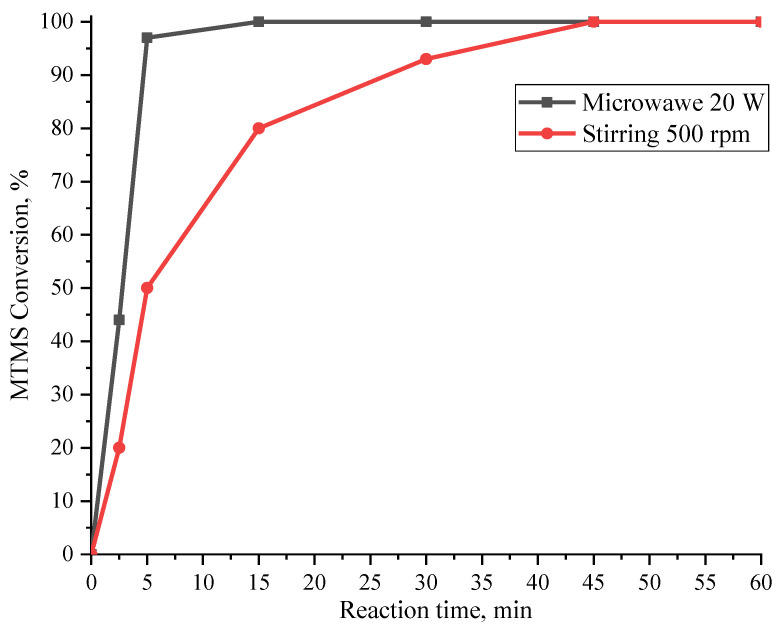
Curves of monomer conversion versus time for MTMS hydrolytic polycondensation under MW radiation (20 W) and without radiation with stirring (500 rpm) at a MTMS/H_2_O molar ratio of 1/3 and at 30 °C.

To confirm the effectiveness of the use of MW radiation as a method for intensifying of the MTMS hydrolytic polycondensation, a comparison of the processes by a MTMS/H_2_O molar ratio of 1/3 and at 30 °C dynamics under MW radiation (20 W, Samples 4, 11–15, Table 1) and without MW radiation with stirring on a magnetic stirrer (500 rpm) was made. Plots of monomer conversion during these processes are shown in Figure 6.

The obtained data demonstrate a significant decrease in the time required to achieve complete monomer conversion, from 45 min with simple stirring to 5 min with MW radiation. This unequivocally indicates a significant intensifying effect of MW radiation on the MTMS catalyst-free hydrolytic polycondensation.

**Figure 7:**
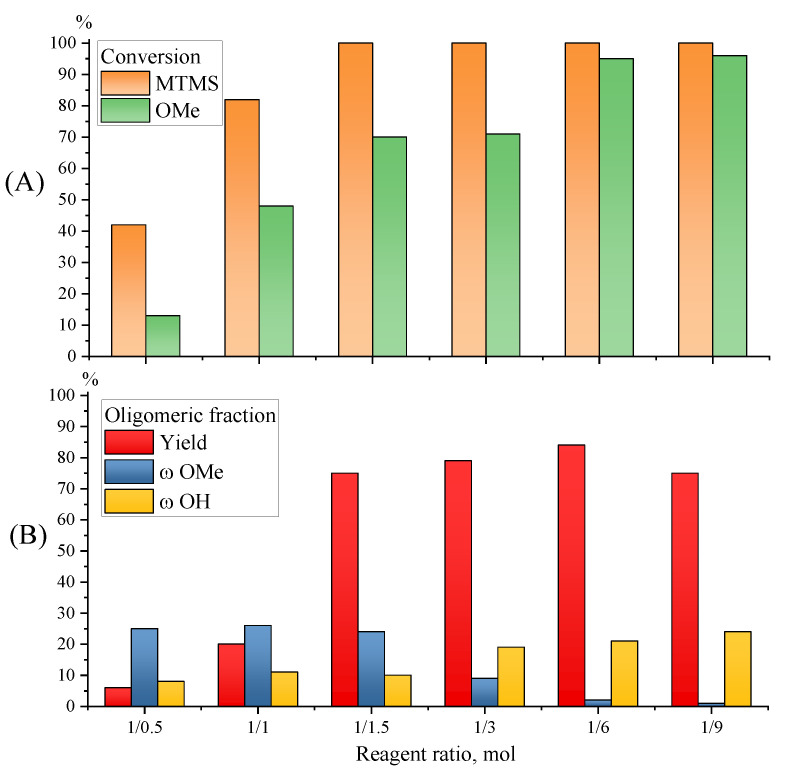
Diagrams of monomer and MeO-group conversions (**A**) and of the yield, MeO- and OH-group contents in the oligomeric fraction (**B**) obtained by MTMS MWHP at different reagent ratios and at 30 °C, 20 W for 90 min (Samples 1–6, Table 1).

The analysis of the resulting products with a power of 20 W at 30 °C for 1.5 h, and a change in the MTMS/H_2_O molar ratio from 1/0.5 to 1/9 (Samples 1–6, Table 1) showed that with increasing water amounts in the reaction mixture, the conversion of monomer increases and already reaches 100% when the ratio of reagents is 1/1.5, with the yield of soluble oligomeric product reaching 75% (Figure 7). It should be noted that during the MTMS hydrolytic polycondensation under ultrasonic radiation, complete conversion of the monomer is observed for the MTMS/H_2_O molar ratio of 1/3 and higher [31], which indicates a greater intensifying effect of microwave radiation.

**Figure 8:**
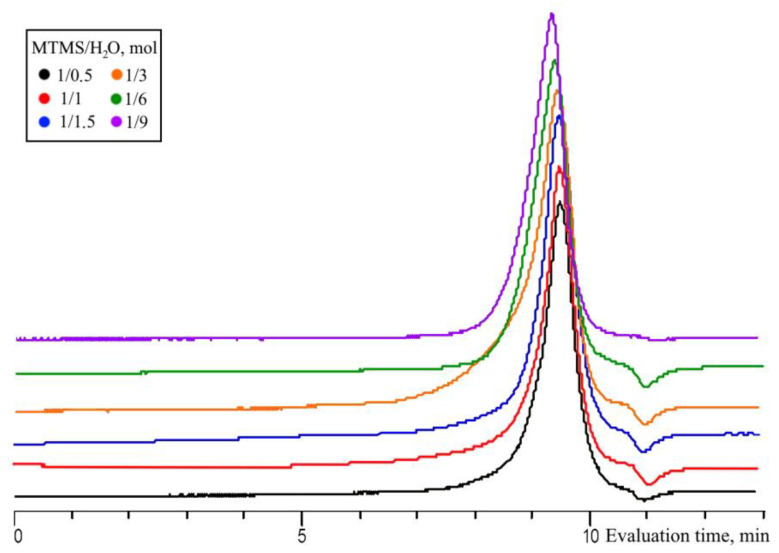
GPC curves for the oligomeric fraction by MTMS MWHP at different ratios of reagents and at 30 °C, 20 W for 90 min (Sample 1–6, Table 1).

The residual MeO-group amount tends to zero with increasing water content in the reaction mixture (Figure 7), while the molecular weight characteristics of the formed oligomethylsilsesquioxanes, according to GPC data, practically do not depend on the ratio of reagents (Figure 8).

Thus, it has been shown that the effect of MW radiation on MTMS hydrolytic polycondensation at different water contents in the reaction mass significantly increases the rate of hydrolysis, but does not significantly affect the rate of condensation, which is confirmed by an increase in the number of OH-groups and an almost unchanged molecular weight of the resulting oligomethylsilsesquioxanes.

**Figure 9:**
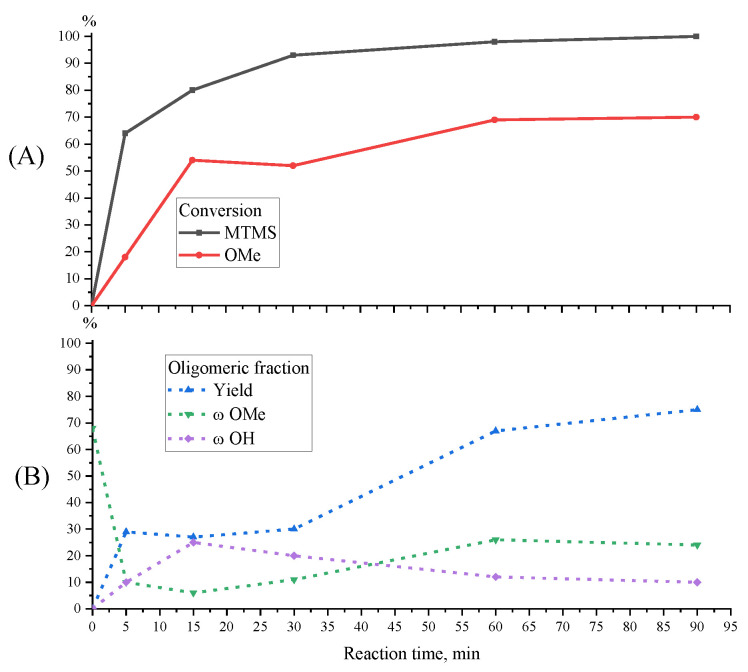
Curves of monomer and MeO-group conversions (**A**) and parameters of oligomeric fraction (**B**) obtained by MTMS MWHP at different radiation times and a MTMS/H_2_O molar ratio of 1/1.5 at 30 °C, 20 W (Samples 3, 7–10, Table 1).

**Figure 10:**
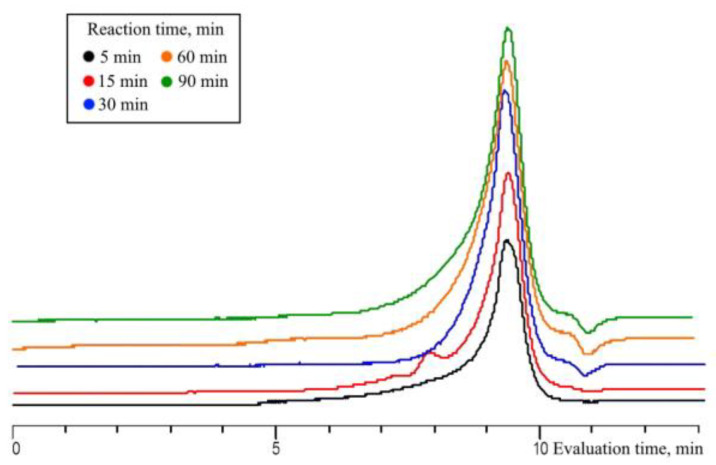
GPC curves of the oligomeric fraction obtained by MTMS MWHP at different radiation times and MTMS/H_2_O molar ratio of 1/1.5, 30 °C, 20 W (Samples 3, 7–10, Table 1).

The influence of the MW radiation time on the MTMS conversion was studied in the range of 5–90 min. Monomer conversion at a reagent ratio of 1/1.5 approaches 100% already after 60 min. In this case, an increase in the yield of oligomeric products up to 67% is observed (Figure 9). The change in the MW radiation time to in the range from 5 up to 90 min has little effect on the molecular weight distribution of the oligomeric product, which remains in the range from 500 to 10,000 Da, with a constant M_p_ (Figure 10). Changing the MW radiation time in the range from 5 min to 30 min has little effect on the molecular weight distribution of the resulting product, which remains in the range from 500 to 10,000 Da, with a constant M_p_ (Figure 10). When the exposure time is increased up to 90 min, an increase in MeO-group content and a decrease in the number of OH-groups in the oligomeric products are observed, which can probably be explained by the reverse esterification of OH-groups by released methanol.

The obtained water–alcoholic solutions of oligomethylsilsesquioxanes did not gel when stored for 2 months at room temperature and for more than a year when stored in the refrigerator. After this time period, the formation of polymethylsilsesquioxane gel occurs. Its composition, calculated by elemental analysis, can be described by the general formula [MeSiO_1.5_][OMe]_0.13_[OH]_0.89_.

The influence of the temperature on MWHP of MTMS was studied at 30 °C, 40 °C, and 50 °C (MTMS/H_2_O = 1/1.5; 20 W; 5 min, Samples 7, 24–25, Table 1). The upper temperature is limited by the boiling point of the released methyl alcohol, the change in the content of which affects the stability of the MWHP process in an open system.

**Figure 11:**
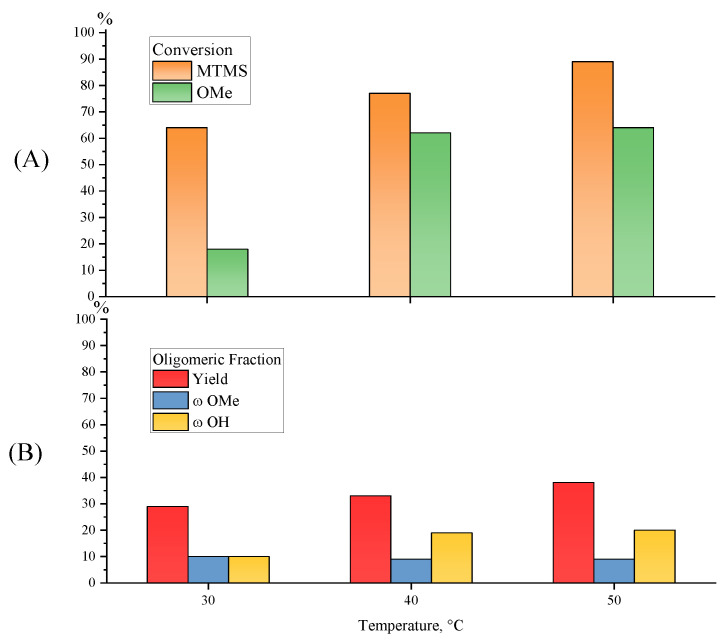
Diagrams of monomer and MeO-group conversions (**A**) and of the yield, MeO- and OH-group contents for the oligomeric fraction obtained by MTMS MWHP (**B**) at different temperatures and an MTMS/H_2_O molar ratio of 1/1.5, at 20 W for 5 min (Samples 7, 16–17, Table 1).

**Figure 12:**
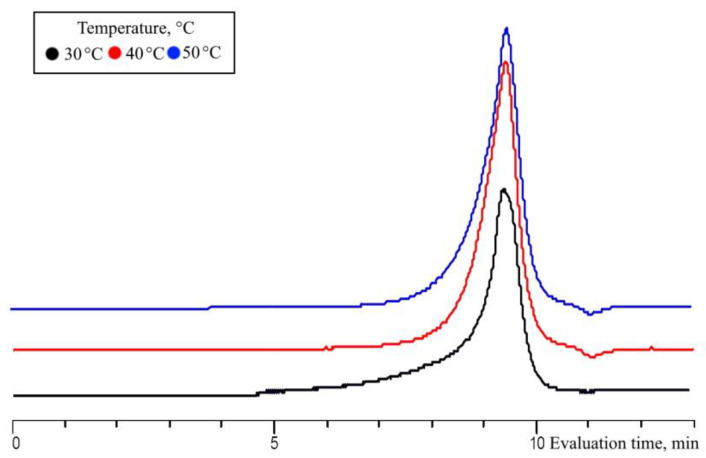
GPC curves for the oligomeric fraction obtained by MTMS MWHP at different radiation times and an MTMS/H_2_O molar ratio of 1/1.5, at 20 W for 5 min (Samples 7, 16–17, Table 1).

The increase in monomer conversion from 64% to 89% and of alkoxy groups from 18% to 64% for 5 min results in an increase in the soluble oligomeric product yields from 29% to 38% (Figure 11) and a slight broadening of the molecular mass distribution of the formed oligomethylsilsesquioxane product with increasing temperature, with all other parameters remaining constant (Figure 12). In summary, it can be stated that increasing the temperature leads to the acceleration of the MWHP process.

**Figure 13:**
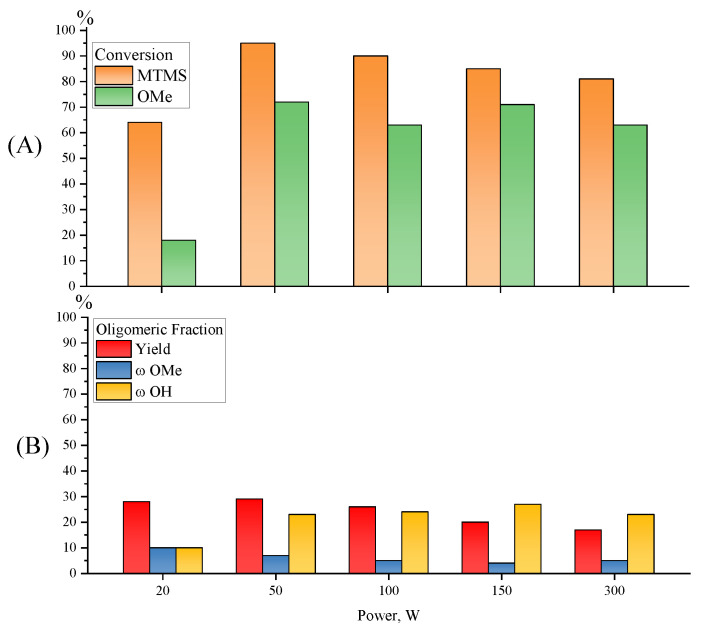
Diagrams of monomer and MeO-group conversions (**A**) and of the yield, MeO- and OH-group contents in the oligomeric fractions obtained by MWHP of MTMS (**B**) at different radiation powers and an MTMS/H_2_O molar ratio of 1/1.5, at 30 °C for 5 min (Samples 7, 18–21, Table 1).

**Figure 14:**
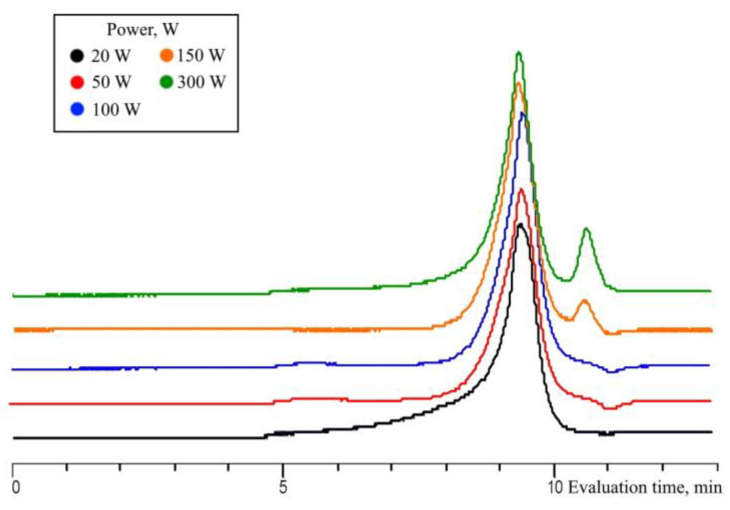
GPC curves of the oligomeric fraction obtained by MWHP of MTMS at different radiation powers and an MTMS/H_2_O molar ratio of 1/1.5, at 30 °C for 5 min (Samples 7, 18–21, Table 1).

The composition of the products obtained by the catalytic-free hydrolytic polycondensation of MTMS under MW radiation insignificantly depend on the radiation power (Samples 7, 18–21, Table 1; Figure 13 and Figure 14).

Increasing the radiation power from 20 to 50 W at a reagent ratio 1/1.5 for 5 min of exposure leads to an increase in monomer conversion from 64 to 95% (Figure 13). However, a further increase in power up to 300 W leads to a gradual decrease in monomer conversion from 95 to 81% and an insignificant change in yield of the oligomeric fraction of products from 29 to 17%. The decrease in monomer conversion with a significant increase in radiation power may be related to the operating principle of the MW system. The point is that the higher the radiation power, the faster the system reaches the target temperature, which leads to an automatic reduction in the radiation power to some average value sufficient to maintain the temperature. That is, the higher the initial radiation power, the shorter the duration of its exposure at a given power, which, in turn, leads to a decrease in monomer conversion. Thus, the optimal values of MW radiation power in the processes of catalyst-free hydrolytic polycondensation of MTMS, leading to the complete conversion of the monomer, are in the range from 20 to 100 W.

## 4. Conclusions

The results obtained show that the use of MW radiation to activate the catalyst-free hydrolytic polycondensation is quite effective. In 5 min at 30 °C and 20 W of MW radiation of reagents in a monomer/water ratio of 1/3, 96% monomer conversion is achieved (Sample 12, Table 1). Within a short time (90 min) with excess water, a high conversion of MeO-groups (more than 90%) is achieved (Samples 5–6, Table 1). Increasing the process power from 20 to 50 W at 30 °C makes it possible to increase both the monomer conversion from 64 to 95% and the residual MeO-group content from 18 to 72% for the stoichiometric ratio of reagents in 5 min (Samples 7 and 18, respectively, Table 1). The maximum yield of oligomethylsilsesquioxanes (75–85%), non-volatile at T_b_ > 85 °C/1 Torr, is achieved with an MTMS/H_2_O reagent ratio of 1/6 (Sample 5, Table 1) at a temperature of 30 °C and microwave radiation with a starting power of 20 W for 90 min.

In addition to technological simplicity, this process makes it possible to obtain a completely soluble polymer with reproducible and controllable characteristics. Depending on the process conditions, homogeneous water–alcohol solutions of oligomethylsilsesquioxane with a concentration of 20 to 50 wt.% can be obtained. The content of OH-groups in the obtained oligomethylsilsesquioxanes can be controlled from 10 to 30 wt.%, and the molecular weight of oligomers from 1000 to 600 Da. The obtained oligomers are of interest as surface modification agents of various nature. 

## Figures and Tables

**Table 1:** Reaction conditions and composition of MTMS MWHP products.

Sample	Reaction Conditions				Product Characteristics						
	Reagent Ratio MTMS/H_2_O, mol/mol	T, °C	Power, W	t, min	MTMS Conversion, %	OMe-Group Conversion, %	Oligomeric Fraction				
							Yield, %	M_p_ (GPC)	[MeSiO_1.5_]/[OMe]/[OH] (^1^H NMR Data)	Content of Groups (wt.%)	
										OMe	OH
1	1/0.5	30	20	90	42	13	6	400	1/0.69/0.44	25	8
2	1/1	30	20	90	82	48	20	600	1/0.77/0.60	26	11
3	1/1.5	30	20	90	100	70	75	850	1/0.68/0.52	24	10
4	1/3	30	20	90	100	81	79	900	1/0.23/1.00	9	19
5	1/6	30	20	90	100	95	84	950	1/0.06/1.06	2	21
6	1/9	30	20	90	100	96	75	1015	1/0.03/1.24	1	24
7	1/1.5	30	20	5	64	18	29	600	1/0.25/0.47	10	10
8	1/1.5	30	20	15	80	54	27	600	1/0,17/1,38	6	25
9	1/1.5	30	20	30	93	52	30	700	1/0.29/1.08	11	20
10	1/1.5	30	20	60	98	69	67	800	1/0.74/0.65	26	12
11	1/3	30	20	2.5	44	10	32	900	1/0.94/0.83	30	14
12	1/3	30	20	5	96	76	48	940	1/0.13/1.21	5	23
13	1/3	30	20	15	97	78	68	915	1/0.16/1.09	6	21
14	1/3	30	20	30	98	75	74	930	1/0.23/1.40	8	25
15	1/3	30	20	60	100	81	77	920	1/0.24/1.27	9	23
16	1/1.5	40	20	5	77	62	23	915	1/0.24/1.07	9	20
17	1/1.5	50	20	5	89	64	38	860	1/0.23/1.00	9	19
18	1/1.5	30	50	5	95	72	29	870	1/0.18/1.25	7	23
19	1/1.5	30	100	5	90	63	26	890	1/0.14/1.33	5	24
20	1/1.5	30	150	5	85	71	21	900	1/0.10/1.54	4	27
21	1/1.5	30	300	5	81	63	17	975	1/0.13/1.21	5	23

## Data Availability

Data presented in this study are available on request from the first author.
